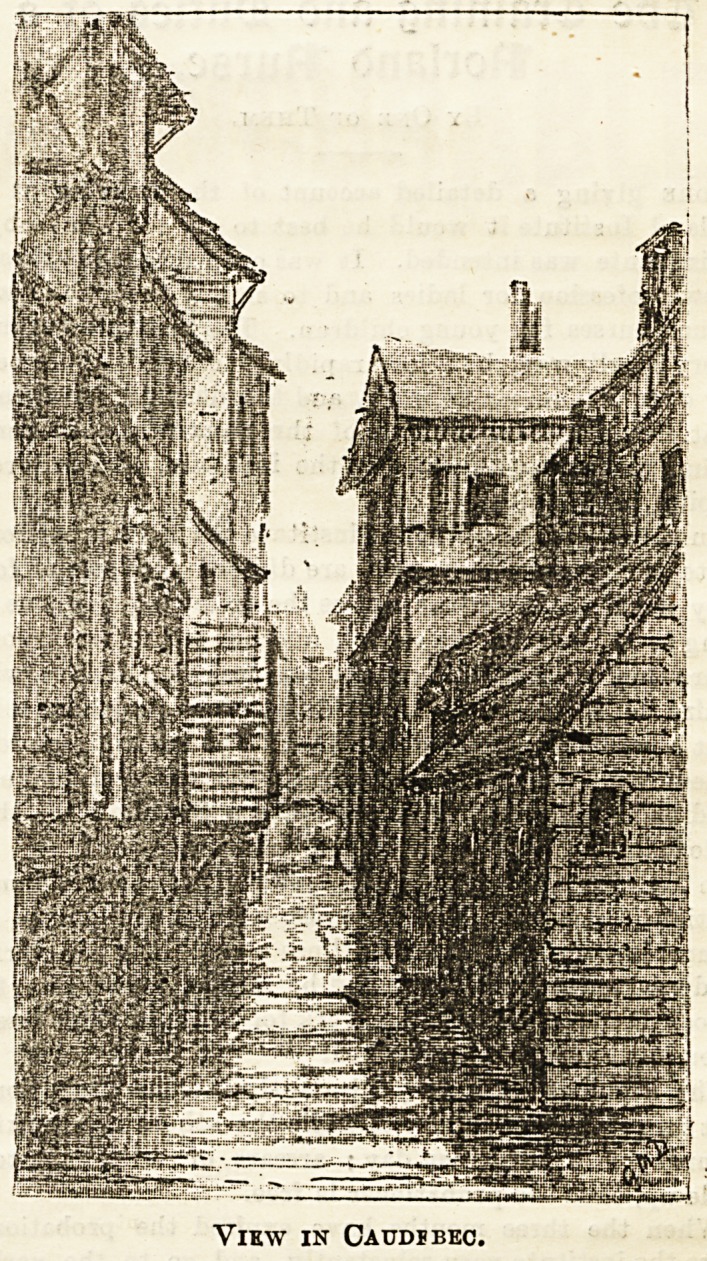# "The Hospital" Nursing Mirror

**Published:** 1899-03-25

**Authors:** 


### "The Hospital" Nursing Mirror.

Being the Nursing Section of "The Hospital."

[Contributions for this Section of "The Hospital" should be addressed to the Editor, The Hospital, 28 & 29, Southampton Street, Strand, London, W.C., and should have the word "Nursing" plainly written in left-hand top corner of the envelope.]

### News from the Nursing World.

### PRINCESS CHRISTIAN AND DOMESTIC NURSING.

In bygone days the care of the sick devolved on their relatives and friends, assisted, as a last resource, by—Mrs. Gamp. Then, when the marvellous results attendant upon trained nursing became known, when the fact was recognised that nurses must be taught as well as "born," and due provision was made for their instruction, then the skill of our grandmothers was dubbed old wives' quackery, and forgotten. But minor ailments are many in comparison with the number of graver maladies, and there is no reason why the friends should entirely hold aloof. The Princess Christian has ever encouraged the spread of a knowledge of domestic and elementary nursing. Amongst her many engagements she has always found time to distribute the medallions and certificates awarded to the successful students of the Norwood centre of the St. John Ambulance Association, of which she has been President since its foundation seven years ago. On the 16th inst., accompanied by Miss Loch and Major Martin, she met the members of the association at the Concert Room of the Crystal Palace and bestowed about four hundred of these awards. Mr. C. E. Tritton, M.P., chairman, received Her Royal Highness, and mentioned, in thanking her for her presence, that the institution was in a very flourishing condition. Lord Knutsford reiterated the thanks of the association for her visit, and expressed those due to her for the warm interest she has ever displayed in its welfare.

### ARMY NURSES.

Captain Norton's question to the Under Secretary of State for War anent the "exact training in months and years of the army nursing sisters and of the army orderlies" elicited the following reply. We quote from "Parliamentary Intelligence" in the *Lancet*: "An army nursing sister is required to have had three years' training in a civil hospital and six months' probation in the army nursing service before her appointment is confirmed. A private in the Royal Army Medical Corps receives theoretical and practical instruction at the depôt at Aldershot for about five months. He is then attached as a supernumerary to a large military hospital to learn practical nursing and ward work, and he remains there until he is considered efficient. His education, however, is systematically continued throughout his service with the colours." It is to be observed that this is not a question of the superiority of male or of female nurses, but a Parliamentary statement of the fact that a training that would be insufficient to qualify a woman for the post of superintendent nurse in a workhouse is considered adequate to fit a man to nurse our soldiers. Pushing the statement to its logical conclusion it amounts to this, that what is not good enough for our paupers will do for our soldiers. It has never been disputed that a woman's aptitude for nursing is greater than that of a man, and it must be therefore acknowledged that the candidate less liberally endowed by nature requires the more careful training. It is to be regretted that male probationers cannot obtain systematic instruction in nursing. For elsewhere, as well as in the army, there is not only a demand for male nurses, but there are certain maladies in which it is imperative to have them. The thought that precious lives, spared in the vicissitudes of battle, may be sacrificed to inadequate nursing is intolerable. Women are no doubt out of place in the battlefield, but common sense, whilst acquiescing in this, will nevertheless insist that the men who do nurses' work shall at least be as well qualified in nursing as a trained nurse in a workhouse infirmary.

### SOCIETY OF TRAINED MASSEUSES.

On Friday, March 10th, the Society of Trained Masseuses held its annual meeting at the Trained Nurses' Club, Buckingham Street, Strand. A large number of members were present, and a good deal of important business was satisfactorily got through. The secretary's report was most interesting, and the record it contained of the progress made by the society very gratifying to those present. The society is now in the fourth year of its existence, and numbers 150 members. A social tea formed a pleasant ending to this successful gathering.

### REBELLIOUS NURSES.

It would be vain to deny that there is a rebellious and gainsaying spirit abroad, or to imagine that nurses are exempt therefrom. We have letters from nurses asking us if they can be compelled to wear uniform, or to obey this or that rule. We hear of resignations being sent in by the dozen when regulations do not fit in with the will of those whom they are designed to govern, and from all sides we have emphatic evidence that both Jack and Jill consider themselves as good as either master or mistress. Loyal service is becoming a thing of the past, yet the truth remains—obedience is better than sacrifice; and above all is it so in dealing with sickness. To be obedient implies self-control, patience, good judgment, nice feeling, and unselfishness, and these are the qualities one seeks in a nurse. It is bitter to those who would reverence the nurse as a ministering angel to learn that there are fully-trained and clever nurses who avoid any institution where there is the slightest restraint, and that the use they make of their liberty is to indulge in late hours, rounds of amusement, and other pleasures which, if not questionable, are certainly curiously out of harmony with the highest ideals of the nursing life. The discipline in the Army and Navy turns hobbledehoys into smart soldiers and sailors. Strict discipline in training-schools produces the finest nurses, and obedience to a high ideal of duty and to those set in authority educates and develops noble characters. There are those who say that the character of the modern nurse has deteriorated. This is, let one hope, a libel, for as naughty children ever make more noise than good ones, so more is heard of the reckless nurse than of her who wearies not in patient well-doing. Yet it is never unseasonable to show up the root of bitterness, and rebellion against rightly-ordained discipline is a plant that can only bring forth thorns.

### LONDON TEMPERANCE HOSPITAL.

On Thursday, the 16th inst., the Duke of Westminster opened an additional ward at the London Temperance Hospital. It contains twelve beds, and raises the number of beds in use to one hundred. The Prince of Wales's Fund contributed £500 towards the cost, and the gentleman after whom the ward is named the remainder.

### IRISH DISTRESSED LADIES' FUND.

We have before us the triple balance-sheets of the Irish Distressed Ladies' Fund, namely, of the London, Scottish, and Irish branches. There are many and various ways in which the fund helps distressed gentlewomen. It gives supplementary pensions by which the recipients are enabled to secure the necessities of life—grants for emergencies, grants towards the education of children, whom it also aids sometimes by securing their admission on the foundation of schools. The society also organises the sale of needlework, and helps members to obtain employment. The amount of work done is enormous, and the attendant expenses heavy, as must always be the case in helping people to help themselves. But the object is a truly charitable one, and touches, perhaps, the most pitiable class of the poor—namely, those possessing refined tastes, and often not possessing the knowledge of how to make a little go a long way.

### THE LONDON SPECTACLE MISSION.

The London Spectacle Mission is one of those agencies which pursues a course of unostentatious usefulness. Its aim is to supply really poor folk whose sight is failing a pair of good suitable spectacles. The income last year, with the balance of the previous 12 months, was £127, and the expenditure £67. The hon. secretary is Miss C. Waring, 197, Sutherland Avenue, W., and there is a branch of the society working in East London at 317A, Mile End Road, E. During 1898 there were 938 applicants for the spectacles, and, when necessary, special lenses were procured.

### FOR THE HOMELESS CHILDREN.

The president of the National Refuges for Homeless and Destitute Children (the Earl of Jersey) took the chair at the annual meeting held at Exeter Hall on March 22nd. Bishop Barry and the Rev. George Hanson addressed the meeting on behalf of the charity. No fewer than eleven homes, schools, &c., including the training-ships "Arethusa" and "Chichester" are supported by the society, of which the office is 164, Shaftesbury Avenue, London, W.C. Over fourteen thousand boys and girls have been trained in domestic, agricultural, and mechanical employment since their foundation in 1843. The expenditure last year was £21,231, and a deficit of more than £1,900 as well as a mortgage on the London Home has to be faced, for the liquidation of which an urgent appeal is made to the public.

### IN MEMORIAM.

The Bishop of Rochester on Tuesday, March 21st, at half-past twelve, unveiled and dedicated a reredos in St. Thomas's Hospital Chapel, which has been erected in memory of the late Sir Henry Doulton by his son. The work is by Tinworth.

### ANNUAL MEETINGS.

There is a plethora of annual meetings just now. Important and interesting as many of them are individually, their records are not infrequently monotonous to the general reader; and therefore we merely chronicle one or two of the more prominent events at the following nursing institutions. On April 1st the Bristol Nurses' Institute changes its lady superintendent. Miss Nolan retires with the cordial good-will of those with whom she has worked, and Miss Annie Thompson-Hill succeeds her. The training of probationers will be discontinued, and for the future nurses holding three-year certificates of training will bs engaged. The salaries will range from £30 to £35 a year, with an added bonus of 10 per cent. on their earnings. The present staff numbers 37. New rules have been drawn up for the guidance of the committees, officers, and nurses.—The Marchioness of Zetland took the chair at the annual meeting of the North Riding Rural Nursing Association held recently. It is satisfactory to note that an adverse balance of 12s. 6d. for 1897 was last year transformed to one of £33 in favour of the society. The County Council Diamond Jubilee Committee has remitted to the society a sum of £172, the balance of the fund raised for the celebration of this Jubilee. It has been reserved for special purposes.—The Mayor of Bangor, in moving the acceptance of the annual report of the Bangor Institute for Trained Nurses, pointed out that the only unsatisfactory feature was the adverse balance of £160, exclusive of the £60 still due to Lady Penrhyn on account of her loan. The Archdeacon of Bangor, in seconding the motion, asked half-crown subscribers to increase the amount of their contributions. Lady Penrhyn was re-elected president, and Miss Pritchard hon. secretary.—The Earl of Sandwich presided at the annual meeting of the Huntingdon and Godmanchester District Nurse Association. The report showed a balance of £30 in hand, and an increase of subscribers contributing 1s. each. A regulation was passed requiring the nurse to give six weeks' notice before leaving. A motion of sympathy was recorded at the death of Miss Simson, the late hon. secretary.

### SHORT ITEMS.

The Corporation of Edinburgh gives an "At Home" every year to the nurses engaged in the hospitals and institutions of the city. About five hundred and fifty guests were present at that which took place on the evening of the 15th inst. The Chief Magistrate (Bailie Kinloch Anderson) received the guests, and good musical, vocal, and dancing programmes were provided for their entertainment. The proceedings ended before midnight.

### Hints on the Home Nursing of Sick Children.

By J. D. E. MORTIMER, M.B., F.R.C.S., formerly Surgical Registrar, &c., at the Hospital for Sick Children, Great Ormond Street.

(*Continued from page* 253.)

SURGICAL DRESSINGS—OPERATIONS—ECZEMA—RINGWORM—VACCINATION—HERNIA—NOSE BLEEDING.

WHEN surgical dressings have been applied restraint is often required to prevent the child hurting itself by restlessness, and if so must be effectual—a half-hearted attempt is worse than none. Sandbags, splints, &c., are used as with adults. To prevent interference with wounds, &c., the hands may be muffled, or the arms confined to the body with a broad bandage or towel, or by putting on the clothing over them; the hands may also be kept from the head by putting on the elbows light splints or a plaster of Paris casing so that the child cannot bend them. If the recumbent position is necessary, a piece of webbing should be put across the bed just below the pillow and fastened to the bedstead on each side; another piece should be cut the width of the chest, and to the ends of this should be attached two armlets (like short broad straps), padded and covered, with buckle fastenings; these are passed through the armpits, underneath the webbing on the bed, and over the shoulders to the front. If necessary the trunk may be further confined by a broad band passing over the pelvis and fastened on each side to the bedstead, but there must be no pressure on the abdomen or chest. The cot or bedstead must, of course, be of suitable size and the mattress firm, with a small pillow fitting into the nape of the neck. A cradle may be needed to take the weight of bedclothes off the feet. There must be no propping up in bed for meals or otherwise.

The child must be rolled on to its side for washing, bed-making, and for introduction of the bedpan, or in some cases it may be better to use a "slipper," slightly lifting the pelvis. In special cases it may be necessary to arrange short mattresses or pillows, so that there is a break or interval into which may be slid a bedpan, absorbent material, or whatever is necessary. For spinal, hip, or similar cases there should be a bed tray on short legs (or running on the side rods of the crib), and kindergarten or other occupations encouraged rather than much reading or being read to. Whenever possible the child should be taken into the open air by being wheeled in a couch or carried out in a light tray provided with straps.

### OPERATIONS.

The usual preparations must be made beforehand. Care must be taken to guard against chill, both during its performance and afterwards, by means of hot-water cushions or bottles, warm socks, &c. When recovering from its effects no attempts should be made to sit the child up or "rouse" him in any way. It must be explained to relatives that the child must recover gradually from the effects of the anæsthetic, and that a long sleep in a quiet, darkened room is the one thing desirable. No food should be given for two or three hours, as it will only be likely to cause sickness. If the child does not seem to be rallying, or if there are any signs of hæmorrhage or collapse, such as steadily-increasing pallor, the surgeon should be at once informed. The horizontal position must be maintained, external warmth applied, and an enema containing brandy given (unless there is hæmorrhage or possibility of such). He should also be told if there seems to be severe pain, as this has a most depressing effect on the system, to say nothing of other considerations. It must be remembered, however, that when the effects of the anæsthetic are passing off there may be a good deal of crying, which does not mean conscious suffering. Do not let the child sit up suddenly when allowed to do so for the first time, as this is very apt to cause faintness even some days after a small operation.

A few remarks may now be made on some ailments very common amongst children which may be met with in district nursing or in those subject to more serious disorder.

### ECZEMA.

Scabs must be well soaked in oil, and if on the head removed by passing underneath them the blades of scissors (with rounded ends) and snipping through the hairs. It is no use to apply any dressing over a scab. Thin gruel should be used for cleansing unless a special soap is ordered. Ointment for the face should be applied on a mask of thin "butter muslin," in which holes are cut for eyes, nose, and mouth, and pieces must be tucked into the folds behind the ears. The nails must be clipped close, and if the child scratches or rubs the hands must be confined in some way, as anything more than gentle patting adds to the irritation, and the matter is spread about to infect fresh places.

### RINGWORM.

Careful cleansing of the surface, followed by thorough and persevering application of remedies are the essentials of success. A light, washable cap should be worn (for the sake of others), and of course the utmost care taken in regard to separation and disinfection of towels, brushes, &c.

### VACCINATION.

The vesicles should on no account be poulticed or even washed, but kept dry and protected by wood wool or similar dressing. Scratching must be prevented. Some boric acid and starch may be dusted on if there is much irritation.

### HERNIA.

If a spring truss is used the nurse should see that it fits fairly closely without bearing too much on any one part, and that it effectually keeps up the bowel; if this comes down the truss is worse than useless. It should be covered with some soft material which may be burnt when soiled. For bathing one must be used covered with gutta-percha tissue or made of rubber, and whenever changing, the nurse or an assistant must be very careful to keep a finger constantly over the weak spot, which may be felt at the middle of the groin. The skin should be carefully dried and powdered and cotton-wool tucked in where necessary. For a navel rupture or umbilical hernia a disc of cork or a large silver coin should be wrapped in lint and fastened with plaster. Belts are of no use. If prolapse of the bowel occurs the child must be laid on its side with a pillow under the hips, and the part that comes down oiled and carefully pushed back and the buttocks kept together for a time with the hand or a broad piece of strapping. Constipation must be guarded against, and the child not allowed to sit too long and strain.

### NOSE BLEEDING,

Apart from injury, may occur at the onset of acute disease, in whooping cough, and other disorders. The child should sit upright and not hang the head over a basin; there should be nothing tight round the neck. The room should be cool. A sponge dipped in cold water should be applied to the nose, and iced water or hazeline may be snuffed up or syringed into it. The child must not pick nor blow the nose when the bleeding has stopped. In case of operation in this region it is well to provide red cloths or handkerchiefs, as a little blood makes a great show on a white one and causes needless alarm. Blood from the back of the nose may run down behind the palate, and, being coughed or vomited up, may seem to come from the lungs or stomach.

Foreign bodies (buttons, &c.) pushed into the nose or ear will, as a rule, do very little harm if left alone until a surgeon can extract them, whereas by attempts at removal the most serious mischief may be caused. If a child chokes the mouth should, if necessary, be forced open with a spoon or something similar, and the finger pushed well to the back of the throat and round the root of the tongue, when the substance can often be dislodged.

### How to Start a County Nursing Association.

By Lady Baker.

### II.—ORGANISATION.

In trying to work a county nursing association, one of the first difficulties which will beset the organising secretary will be the opposition of some, and the unreasoning energy of others. To give an instance of the first. Some town in the country, say, of three or four thousand inhabitants, on whose support the committee specially counted—let us suppose some ancient borough—will, directly the idea of a county nursing association is mooted, absolutely refuse to have anything to do with it, and immediately resolve on setting up a nurse for itself, on some manifestly selfish lines of its own.

In vain you urge that "union is strength." Your mouth is closed by the indisputable answer, that this is an excellent argument in the hands of the weakest of the community, but not in those of the stronger and self-sufficing ones.

You then appeal to their feelings of pity for the weaker ones—the outsiders in the neighbouring villages, who can boast of little or no subscriptions, but much need. Strong in the patronage of the local magnate, whose main contribution they have already taken care to secure, regardless of the wants of the same neighbouring villages, which have an equal claim on it, they reply, "Let each look out for himself," and so their independent organisation, instead of being a centre of usefulness for all, a rallying point in the district for the scattered hamlets around, and a means of training less skilled workers for poorer parishes, remains a self-centred block, in the midst of linked communities, working on wider and more unselfish lines.

Do not despair, but wait for time to prove and test their work, and for a central Home, and organised county fêtes, to make them feel a "little out of it." Turn your attention from these, and such as these, whose apparent thirst for information is not in order that they may join you, but only to make use of your experience, and give your time and attention to those who are really desirous of working hand-in-hand with you as part of a coherent whole, and not as isolated units. Theirs may be the energy which may possibly need direction. An instance will probably before long come under your notice, in some such way as this: The energetic and capable wife of some neighbouring clergyman, with business capabilities far beyond her narrow sphere of action, will resolve to start a nurse for her own village—say, of 500 souls. She collects the funds in a burst of enthusiasm, aided by her great friend, the squire's wife, a branch is formed and affiliated to the county association, a nurse started, and all promises fair. A gentle hint offered by the county secretary that a nurse between her own village, and two neighbouring ones of similar size, also belonging to the same squire, would be amply sufficient, has not been received with much favour, the neighbouring parsons' wives being declared "impossible to work with" (no wonder, poor souls, with ten children apiece!) Well, the branch prospers till a healthy summer comes, with no illness, and having little or no occupation, the lonely nurse is tempted to indulge in a little local gossip, for she, after all, is but human; when, heigh presto! she is quickly replaced by not only one, but by two or three other active workers in succession, each one to retire in her turn, on the same plea of "nothing to do."

For every evil under the sun,
There is a remedy, or there is none.

In this case there are two. When a parish is too small to afford work for a nurse, let two or three villages club together and share the nurse between them, having each a vacant room ready to receive her when sent for, and each aying her in proportion to the work done in their own village; or, if preferred, per ratio of population. Or another way to arrange is for each of the three villages to contribute in proportion, to the cost of keeping a nurse at the central county home, sending for her when wanted, and, when not wanted, letting her earnings elsewhere be credited to their account towards the diminution of her cost.

When I first started as secretary of a county nursing association, now nearly seven years ago, I believed that work could easily be found for a nurse by any one village keeping her, and other neighbouring villages hiring her, when not wanted, nearer home; but I have frequently found that local jealousy renders this at times almost impossible. It certainly is a fact that in whatever village a nurse resides, that village gets the most of her services. I have sometimes thought that it would be a good plan that in all towns over three or four thousand inhabitants, two nurses should reside—one an urban district nurse, supported by the town itself; and the other a rural district nurse, maintained by the rural districts, and available for them at all times. Should there be no demand for the latter in the rural districts at any time, she might take extra and paying work among the well-to-do classes, the payment of which should go towards her expenses. A small charge for her services should be made to each village alike.

Let us now turn to the vexed question, the medical committee. I am convinced that no county association can thoroughly deserve the name or really prosper, unless it has a medical committee, to whom all purely professional matters should be referred. The lady enthusiast and the amateur philanthropist are, as a rule, sublimely ignorant of medical etiquette, not to say the meaning of medical terms, and it is advisable that some slight guidance should be accepted from the leading medical men of the county, in avoiding professional pitfalls, and in meeting, let us say, the slightly superior smile of some youthful nurse, secure in the latest "ologies" of the dissecting-room. As to whom should form the medical committee, that would probably be best decided at some such meeting as that of a local branch of the British Medical Association, who would most likely be able to select the best county representatives, without arousing professional jealousy, from which, alas! neither the army, the navy, nor the medical profession is altogether exempt.

A little tact and courtesy will, however, easily avoid shipwreck in this direction, and, as it is a question in which both doctors and nurses are pre-eminently concerned, it is but fair that their side of the question should be represented on the organising body. To this purpose, it is also generally advisable to have one or two trained nurses, such as matrons or ex-matrons of county institutions on the central committee, as their practical knowledge, from the nurses' standpoint, will often save many disappointing experiments. Unless you secure in some such ways as these, the hearty co-operation of the professions most concerned, as likely as not your organisation will fall still-born, or die out after a little period of fruitless effort. The general practitioner has to be convinced, not an easy task, that the influx of a body of trained nurses into the county will not injure their practice. The nurses have to be convinced that their interests will be safeguarded, and no unnecessary rules imposed upon them. The public have to be impressed with the fact that skilled nursing is worth its cost; a fact which in out-of-the-way country districts is warmly debated. All these will test the faith, the perseverence, and the patience of the governing body, and only time will be able to give an answer to the many who, in their heart of hearts, re-echo the cry of the wilful would-be philanthropist, who was heard to exclaim, "Organisation! I hate organisation!" True, my lady, but without it our boasted civilisation would become a chaos.

### The International Congress of Women Workers.

THE NURSING SECTION.

It is time Lady Aberdeen, who is president of this Congress, again took up her residence in London, for unless she assumes the active direction of affairs without delay the Congress is likely to prove a failure. When arrangements were made for holding the International Congress a nursing section was resolved upon which should be representative of the nursing profession. Unfortunately, in Lady Aberdeen's absence this so-called section was captured by a lady who has made herself notorious in such matters, and who proceeded to hold the meeting at her private house, and generally to ignore all who were really representative of nursing in the United Kingdom. In consequence of this a representative Matrons' Sub-Committee was formed, who sent a deputation on January 23rd last to Lady Aberdeen, which presented a written protest against the unsatisfactory arrangements which had been made for convening the nursing section. It suggested that it should be made into a subsection, and be placed in the hands of the profession to work. It was pointed out that before the preliminary arrangements were made the heads of the nursing profession should have been consulted, that a suitable representative, who would command the respect and confidence of the whole nursing profession, might be selected. To put at the head of the nursing section a lady who had had no connection with any training school for nurses for more than 10 years, and who did not represent any large body of nurses at the present time, must prove fatal to the success of the nursing section.

In the result the Sub-Committee of Arrangements for the Congress determined that the meetings of the International Congress at which nursing and medical questions are concerned shall be confined to papers written by foreigners, and that no English speakers shall be permitted to take part in the discussion. It was further determined that the meetings of the nursing section should be presided over by a lady unconnected with either the nursing or the medical professions. How far Lady Aberdeen is responsible for this extraordinary decision, which amounts in effect to a declaration that nursing is a matter of no interest to English people, and that only foreigners understand anything about it, we cannot say. We are perfectly confident, however, that if this decision is to stand it must seriously affect the finances of the International Congress and largely destroy public interest in its proceedings. How Lady Aberdeen, as an Englishwoman, can remain president of a Congress which declares, through the Sub-Committee of Arrangements, that English nursing and English speakers on nursing and medical topics are not entitled to be heard or considered passes our understanding. We are not surprised that the Matrons' Sub-Committee, as representing the nursing profession of the Empire, should have resolved to take no efficient part in the meetings of the nursing section, and to leave the International Congress of Women Workers severely alone. We hope, unless wiser counsels prevail, that Lady Aberdeen, as a loyal subject of Her Majesty, and one who has taken an active part in English nursing matters, will decline to be further connected with the International Congress of Women Workers.

### Pension Fund Nurses.

MISS BURNS' WEDDING GIFT.

We have to acknowledge the receipt of further contributions, viz.: From Nurses Rennie, E. C. Wilson, A. Bryan, A. S. C., and J. Gibson. Any other intending contributors are positively informed that no further contributions can be received after the 28th inst. The list was closed some time ago, and we have only included the further contributions we have done at the earnest request of contributors, some of whom were abroad when our preliminary notices appeared.

### Hospital and Home for Incurable Children,

2, Maida Vale, W.

Children thrive best in small homes. It has been proved times out of number that fostering individual cases, or "mothering," is essential, not only to make child life happy, but to develop the child mentally, morally, and physically. If this be the case with healthy youngsters, how much more is it so with those poor little ones whose slender tale of years is full of pain? The good work of the Hospital and Home for Incurable Children, 2, Maida Vale, W., is to gather together into a bright and cheerful dwelling about thirty afflicted babies, and there to nurse them, teach them, and in every way to assuage the sorrows of their lot until they reach the age of sixteen. These children come from the homes of the poorest, some from tenements where poverty is the least evil. On the occasion of our visit it was a pleasure to see the pretty rooms, their occupants, when physically fit, occupied with dainty needlework, bent iron work, and wood carving; to note the deftness of the wasted elf-like fingers, and the hearty delight they took in their own accomplishments. Canon Duckworth presided at the annual meeting, held at the home on the 18th inst. There were also present Lieut.-General Lowry, C.B., the Hon. Treasurer (Mr. E. A. Bonnor-Maurice), the Hon. Secretary (Mr. H. Sewell), Mr. T. Pickering Pick, F.R.C.S. (hon. consulting surgeon), and many other friends and supporters of the institution. Thanks to donations and legacies the balance-sheet is fairly even, but the committee of management would naturally prefer to invest and not to spend the extraordinary income. The figures stand as follows: £882 ordinary income; £1,327 expenditure.

The most anxious problem attendant upon this charity, from whichever standpoint it may be regarded, is the lack of a supplementary home to receive the patients between the ages of sixteen and twenty. There are many homes open to them at the latter age, but the difficulty of finding a refuge for them at sixteen is almost insuperable. The case of Edith Kenney illustrates this. She was received into the home, but at the age of ten, being considered curable, she was sent back to her own people. One of the lady visitors, who had taken an interest in her, after the lapse of about eighteen months, sought her out, and found her being slowly starved to death, the doctor called in declaring that in another fortnight death would have been inevitable. A home was sought for her everywhere, and finally for the next few years this lady and a friend supported her, completing their good work by having her taught church embroidery, by which she will shortly have the power of earning a livelihood. Only once has she visited her father's house since she left it, and on that occasion was disgusted and unspeakably distressed by the squalor of her surroundings, the vile language and dissolute habits which she heard and witnessed. Information respecting the administration of the charity or the admission of patients may be obtained from the hon. secretary or the matron at the home.

### Minor Appointments.

Tolworth Joint Hospital for Infectious Diseases.—Miss Anne J. Ainsworth, who was trained at St. Thomas's Hospital, has been appointed Head Nurse and Superintendent of this institution. She has held posts in the Sanatorium, Cardiff; in the County Hospital, Cardiff; and in the W. M. Hospital, Hampstead.

Isolation Hospital, Nottingham.—Miss M. I. L. Pierce was appointed Sister of the above on February 22nd. She was trained at Salisbury Infirmary, and has held posts in the Monsall Hospital, Manchester, and, as sister in charge, at the Sanatorium, St. Helens.

Infirmary and Dispensary, Bolton.—Mrs. C. M. Crawford has been appointed Night Sister of the above institution. She was trained at the Leeds Infirmary, and was for two years sister of the surgical wards and theatre at the Grimsby District Hospital.

### The Hospital for Sick Children, Great Ormond Street.

### OPENING OF THE NURSES' NEW HOME.

The nurses' new home at the Hospital for Sick Children is, in fact, the old convent of the nuns of St. John and St. Elizabeth. The Order from whom the property was bought carried away their old church with them—a removal which has cost them something like £5,000. The old hospital has been pulled down to the ground floor, and this has been roofed in order to maintain a thorough communication between the Children's Hospital and the old convent, now the nurses' home. One or two rooms have been left. The whole of the premises have been painted, cleaned, and the floors stained, whilst the rooms have been utilised in various ways. The first on the right is a reception-room for the nurses' visitors; the second, the sisters' dining-room; the third, the matron's sleeping apartment and bathroom; a fourth has been fitted up with lavatory basins and divided by partitions to form a couple of extra bathrooms. There are also three single bedrooms, and then the nurses' sitting-room. The sitting-room was the nuns' chapter-room, and is a beautiful apartment, so large that it has the appearance of being low, although this is not the case, as it must be about 12 ft. in height. It is square, panelled all round with old oak, and on one wall some of the lovely old carving given by Sir George Bowyer to the sister in olden times, and now, cleaned and restored, is an adornment to be proud of. The chimney-piece has been put in to match the panelling. Excepting the linoleum-patterned-like parquet on the floor and three small tables, the furniture has been given. The upright grand piano—one of Pleyel's £100—and the rugs that relieve the bareness of the floor are evidences of the chairman's liberality. Friends have given the couch, the comfortable easy chairs, the mirror above the mantleshelf, and the tasteful green serge with which it is upholstered and curtained has been fashioned into shape by the workpeople attached to the hospital. The walls are tinted yellow, a good contrast to the oak. The effect is luxurious, but upon examination it is found to be the result of making the most of a really good room, and the cost has been small. In the basement a similar room is being fitted for a nurses' dining-room. Both rooms look on to the garden, which will be such an acquisition to the little patients as well as to their nurses. This garden has a double advantage; it is not only pleasant in itself, but it gives access to another garden belonging to the hospital garden, of which the entrance had been blocked by extensions. The forty bedrooms, which the home contains, open to the right and left into long corridors having a window at either end. There are fan ventilators over the doors, and blocks are screwed at the bottom of the window frames to ensure the ingress of fresh air at the sash. They are furnished in ash, the suite consisting of a plain substantial wardrobe, a washing stand, and a chest with mirror. The furniture has been supplied by Messrs. Tarn. At present the floors are bare, but for one rug, and the boarding is old and uneven, but they will be covered later, when more money comes in. There was no formal opening. On Wednesday the 22nd, however, the home was open for inspection. The whole is lighted by electricity and heated by hot-water radiating coils.

### Presentation.

On March 14th the nurses and students of Westminster Hospital presented Sister Hollond (Miss F. M. Phillips) with a gold watch, chain, and travelling clock, on the occasion of her leaving the hospital, as a small token of their affection.

### Women's Industrial Council.

### JUVENILE CRIMINALS.

The Rev. W. Morrison, D.D., late chaplain of Wandsworth Prison, gave an interesting lecture last week at 39, Gloucester Square on behalf of the Women's Industrial Council, taking for his subject "The Treatment of Juvenile Offenders." Mr. Morrison gave his audience an account of the various schools, Day Industrial Schools, Truant Schools, and Reformatory Schools, to which youthful breakers of the law are sent, expressing his own conviction, based on long experience, that a prison is the worst place in the world for any young people to be sent to. To send children to gaol was the surest way to turn them into habitual criminals. Children came within the arm of the criminal law from causes individual and social; amongst the former physical weakness and mental incapacity were the most fruitful in bad results, adverse parental conditions being the chief predisposing influences in the latter class. Habitual criminals were generally those who had never been taught a trade, and had been left to themselves in early life; train the children and the supply of habitual criminals was cut off at once. Mr. Morrison dwelt on the importance of proper food for children confined in these reformatory institutions, and the need of finding work for them when they left. It used to be customary to turn them out, literally into the street, without any attempt to make provision for their future, and though things had altered for the better in this respect, Mr. Morrison told his hearers that more voluntary help was much needed in the way of following up the boys and girls leaving schools or prison to begin the world again. Mr. Morrison drew attention to one important matter which shows how far we are yet from grasping the first principles of juvenile training. This is the want of any definite system of teaching trades, so that it is still possible to find a school where the young inmates are kept at the useless occupation of chopping wood, work which fits them for no sort of employment when discharged. He urged the importance of teaching at these schools agricultural pursuits, which would train the boys for working on the land with intelligence, and generally of turning them out equipped for taking up their share of citizenship, especially deprecating the old idea that punishment, simply *as* punishment, would help to solve the problem of how to deal with youthful criminals.

### Appointments.

The Mill Road Infirmary, Liverpool.—Miss Margaret F. Mulvany has been appointed Home Sister here. She was trained at the Royal Infirmary, Liverpool, for three years, and for four years at the Infirmary for Children, Liverpool. For the last two years Miss Mulvany has held the post of senior sister-in-charge of the theatre, out-patient department, and surgical ward at the Royal Infirmary, Wigan.

Wolverhampton and Staffordshire General Hospital.—Miss Annie Tanner was appointed Head Nurse of the female medical wards of this hospital on March 8th. She was trained at the Royal Infirmary, Bradford, and was gold medallist for 1897-8. She has been charge nurse of the female and children's wards at the Rochdale Infirmary since April, 1898.

Highworth and Swindon Union Workhouse.—On the 15th inst. Miss Rose E. G. Nash, who was trained by St. John's House, Norfolk Street, Strand, was appointed Superintendent Nurse of this institution. She holds a midwifery certificate from Queen Charlotte's Hospital.

### Wants and Workers.

Miss Gillett, 2, West Bar, Banbury, would be glad to hear of a pair of crutches for sale, springs under the arm, height 49 inches, for a poor girl.

Nurse Edith, 12, Vale Road, Hermitage Road, Green Lanes, N., would be grateful to any reader who would send a small quantity of old linen for poor patients with bad legs.

Mrs. Monro, Allen, Fearn, Ross-shire, N.B., would be glad of information as to the working of District Nursing Associations, especially in cases where men are on the committees.

### A Book and its Story.

### "SUNSET."

The name of Beatrice Whitby is already known to the public through her authorship of "The Awakening of Mary Fenwick," and, therefore, as a writer, she requires no introduction at our hands. Miss Whitby's latest story, "Sunset,"* the volume at present under review, is the part-history of a girl's life, and it ends in her happy marriage with the man whose strength of purpose and devoted allegiance had stood her in good stead in her hour of need—a man who was neither the creature of her dreams nor the lover of her girlhood.

The plot opens in London, in a West-end household, where Frances Blake is introduced to us. Frances is the heroine of the story, though it is round another prominent character in the book that the tragic interest centres. The girl has just come to settle in town. "She had deliberately chosen to make her cousin's little house in Norton Street her headquarters for awhile; she had done this, as she did most things, of her own free will. She was of an age to judge for herself, and she thought herself capable of counting costs: she was five and twenty years old. For many a year now she had known her own mind, and, unthwarted, had followed its common-place inclinations. She considered herself a lucky woman—she had a clear head, an even temper, as many friends as she needed, and, last but not least, a nice, handy little income of £800 a year, which formed a firm background to an independent spirit."

This fortune above mentioned had been a windfall of recent years, and she, who had a great appreciation of the comforts of life, valued her possession. The girl's life had been an easy one; "she had wished to be comfortable, and she was comfortable." We are told her tastes had not been of any extravagant order, that she was not dangerously good-looking, that she had not been in love since she was 17, and that her strongest sense was common sense. Quite recently Frances Blake's circumstances had undergone a change consequent on the marriage of her sister, her only near relation; and then Wayfield, the home in Devonshire, which the sisters shared, had been shut up, and Frances had planted herself for the time in the little house in Norton Street, "that narrow street which lies so near Belgrave Square that light and air and space are hardly necessities for the Babylonian dwellers therein." "Isabe Beaumont and I get on very well," she had said to her sister, discussing the pros and cons of the future. "I shall put up with her, if she'll have me, whilst you are away. I like Isabel, and I like being in the house with a woman who is as decorative as a beautiful picture." And Mrs. Beaumont had been nothing loth that her cousin should come to her. She and her husband had been suffering from the universal shrinkage of the age. "Their incomes had been modest always, not increasing, as incomes should, with their expenses. Miss Blake knew this, and she had fixed her plans accordingly. "With per cents. and rents and business down to zero," she had written, "don't imagine that I am going to bestow nothing but my society upon you." And so the question was settled in a satisfactory manner to all concerned. The master of the house fell in agreeably with the arrangement. The solitude à-deux in Norton Street was not of the kind the loss of which is grudged. During the long hours that the breadwinner passed in the City, digging and delving with no very encouraging success, it would be well, he thought, that his wife had a sensible woman in the house. "Isabel was amiable and she was fair to look upon, but the domestic hearth had no attraction for her. She lived in what John designated as an 'eternal racketing.'"

Life in Norton Street proved very pleasant to the newcomer, but Frances had an object in coming there other than pleasure, though she had not said so, even to herself. . . . She was only quite certain of one thing—she did not want to go home; she shivered when she remembered Wayfield, and she was only quite certain of one thing, "and the one thing certain was this, she did not want to go home." Among the callers, constant callers at Norton Street, were two men, a Captain Bing and a Mr. George Brand. Round the former the dramatical element of the story centres, and round the latter the romantic. Long ago, in her days of early girlhood, Frances and George Brand had been neighbours; later, something more than neighbours. It was the old story, the suppression of affection for a worldly motive. Frances had higher ambitions, and the two parted. And now, after a lapse of many years, they meet again. George Brand is a widower. He is partner with John Beaumont in a city business. Frances and he become friends again—friends, but nothing more. When George Brand's wife died his heart was buried with her in the grave, and what love was left was centred on the child of the happy union. The girl's feelings and affections are centred on the widower, but she has to content herself with the crumbs which fall, and to be satisfied with esteem and respect from the man who once had offered her his heart. About this time in the development of the story a serious reverse comes to the fortunes of the Beaumonts. George Brand goes to Australia with hopes of averting a final crash, and Frances takes her cousin and her child down to the country, to the place she had never wished to see again. And it is while they were at Wayfield that Captain Bing plays the part of a false friend to John Beaumont, at whose house he had ever been a welcomed guest. He was a cousin of Isabel's, and as children they had played together. Afterwards, in town, when she was married and the mistress of a charming house, he had spent much of his "leave" there, and thus, without premeditation, they had drifted into feeling the necessity for each other's presence. Affairs come to a serious crisis just before the termination of Captain Bing's long leave, when Isabel was down at Wayfield. Isabel in a weak moment yields to the tempter's voice, and she elopes with her cousin. Whilst on the journey a sudden illness lays the woman low, and she dies. Her husband's presence alleviates her last moments, and Isabel Beaumont dies repentant and forgiven.

Added to the terrible tragedy of her sister's end, Frances has other and more mundane cares, which press upon her. Whilst still smarting under the greater sorrow, and, indeed, coming out of that sorrow, came the happiness of her life. The Valley of the Shadow was passed at length, and the woman who had sought for happiness throughout the strong reliant spirit of her girlhood found it after many days in her maturer womanhood, and it came to her, as it comes to many among us, in an all-unexpected guise. "There was something, which she had thought dead, that stirred in her heart, and listened to his voice—

'If she had—well, well, she knew not wherefore,
Had the world nothing she might live to care for?
No second self to say her evening prayer for?'"

And we leave Frances Blake happy.

* "Sunset." By Beatrice Whitby. (London: Hurst, Blackett. 1898.)

### Where to Go.

Dowdeswell's Galleries.—Messrs. Dowdeswell and Co. are now showing at their galleries in Bond Street a collection of water-colours by Mr. E. A. Rowe. The subject is an old garden, and this idea is carried out all through the series. Without bearing the touch of a master hand, these studies possess a certain grace and charm, and should not be missed.

Association of Asylum Workers.—On Monday, March 27th, at half-past three p.m., the annual meeting of this association will be held at the rooms of the Medical Society, 11, Chandos Street, Cavendish Square, W. Sir James Crichton-Browne will take the chair.

### The Abbey Institute.

THE Abbey Institute is a private nursing home designed for the use of gentlefolk who can only afford moderate terms, and to supply fully-trained nurses to the general public. The house is situated in Station Road, a few minutes' walk from Willesden Junction Station. It stands high, and has the benefit of three aspects, east, west, and south. The rooms are lofty and the furnishing adequate and comfortable, whilst the home appears to be thoroughly well kept. Only three patients are received, but they may be medical (not infectious), surgical, massage, chronic, or accouchement cases. The terms are from £2 2s. a week, laundry and stimulants being extras, and are, of course, under their own medical attendant. Especial facilities are arranged for operations, one room is set apart for the purpose, whilst everything that could possibly be needed on such occasions is kept handy. A fair number of patients, resident in town, avail themselves of the conveniences afforded here. Expresses from Euston to Willesden occupy only eight minutes in transit, which is a great advantage in a busy doctor's eyes. The lady superintendent, who undertakes the home nursing, has herself had twelve years' hospital experience. She was trained at Nottingham General Hospital, and immediately afterwards took the post of sister at the Borough Hospital, Liverpool. She then became sister at the Marylebone Infirmary, assistant matron at the Worcester General Infirmary, and matron of the Bradford Nurses' Institute, and possesses excellent testimonials from all these places. Her staff of nurses number eleven, and she requires each to have had three years' hospital training, though not necessarily at one institution only. Their salaries are increased yearly by £2, thus ranging from £31 to £40, with indoor uniform. The charge to patients for nurses is £2 2s. a week, but at a doctor's request a nurse is sometimes sent out at a fee of 30s., but never under that sum. The next step below is to provide a nurse free if she can be spared and the need is urgent. The lady superintendent was one of the first subscribers to the Royal National Pension Fund for Nurses, and encourages her nurses to follow her example.

### A Useful System of Furnishing.

MESSRS. NORMAN AND STACEY (Limited), 118, Queen Victoria Street, E.C., have originated a "System of Furnishing" which will prove convenient to many. Credit is allowed to their customers for varying periods, payment being made by instalments, whilst at the same time, by an arrangement with the Royal Exchange Assurance Corporation, the firm, at its own expense, insures the life of each one of its customers to the amount of his purchase. Where credit is for 12 months only, if the customer dies before the goods are paid for, a receipt in full, no matter how few instalments have been paid, is handed to the next of kin, with a cheque for the amount already paid, while if the period of credit be for more than 12 months, the relations are relieved of all further liability, and in both cases they remain in possession of the furniture. That is to say, if you hire £100 worth of furniture, and die when £80 have been paid, the furniture becomes the property of your next of kin, and the £80 are returned also. A table at the end of the small booklet issued by Messrs. Norman and Stacey, setting forth the advantages of their system, gives the varying amounts of payments and interest, the latter (5 per cent.) being only charged where the credit is for over 12 months. Messrs. Norman and Stacey are not only furniture dealers. They also can supply linen, plate, glass, cutlery, and all the etceteras which go to the complete furnishing of a house, while if they do not happen to have on their own premises the exact article required by a customer an inspection order for the manufacturers' warehouses is supplied. Goods are delivered free of charge by Pickford or other carriers.

### Examination Questions for Nurses.

"NURSE ESSEX" is the winner of the first prize, and "F. I. Evans" takes the second. The answers this time are exceptionally good. Most of the candidates seem to have grasped the difficulties to be grappled with, and to have done so successfully. The winner of the first prize gives two different methods to suit varying circumstances, and thus shows herself to be a woman of resource. Some of the answers show that the writers allow their minds to wander from the subject in hand, and give dissertations upon the manner of administering nourishment to the patient, and also give information as to the right temperature of the bath. Now on neither of these subjects is enlightenment asked for. All that is asked is how best to support the patient's body with the least fatigue. Sketches and diagrams are inadmissible. Candidates are again cautioned against slovenly writing and badly-expressed sentences.

FIRST PRIZE.

The following is my idea of the best plan for supporting a patient with the least possible fatigue whilst undergoing the water cure. A hammock arranged in a bath, the top of which should be slightly raised and an air or water cushion secured to the head of it, serving as a pillow; the patient can then lie comfortably immersed to the chin. Where expense has to be considered, an ordinary strong sheet and plenty of rope would answer the purpose. After measuring the sheet to the desired length, put a broad hem top and bottom or some curtain rings for the rope to go through, but in either case great care must be taken to sew it very firmly. There are many ways by which it could be suspended—from large hooks in the ceiling, or nails might be driven in the woodwork enclosing bath and the rope securely fastened to them at the head and foot. If a portable bath, the rope could be taken over the head-piece of bath and fastened to the wheels, the same applying to the foot.

SECOND PRIZE.

A simple plan is to get a tough sheet and make a broad hem each side. Have two long poles the length of sheet and bath, insert in hem, forming a kind of stretcher. On to the bath outside fasten four strong hooks upside down, one each end, then catch the poles in, the sheet forming a kind of hammock in the bath. You will find this a most pleasant way to immerse in a bath for some hours. The head of the patient can be easily supported against bath with an air pillow.

QUESTION FOR APRIL.

State the method you would employ to make up and keep going a fire noiselessly in cases of brain disease, and also how you would arrange a fire to last all night without attention?

Answers not to exceed six hundred words.

### Our Convalescent Fund.

THE value of our Fund cannot be better shown than by the following letter from "Policy 6,404" to the secretary of the Royal National Pension Fund for Nurses:—

"Can you manage to thank, through THE HOSPITAL, the kind friends who helped me to go to America, and to tell them that the trip has really seemed to have cured me at last of the terrible blood poisoning I had suffered from for eight years? Of course, I shall never be fit for hard work again, but for nearly two years I have had no abscess, so we really think that the disease is eradicated. I am truly grateful to all who helped me, and enclose to you 2s. for the Convalescent Fund, and hope to send you a little from time to time as I can afford it, but I am not strong enough to earn a big salary. Thank you for all the trouble you take for us nurses."

WE have added other contributions of 7s. from M. R., and 3s. from Policy 4,812 to our fund, and we offer our grateful thanks to the donors.

### Travel Notes.

By Our Travelling Correspondent.

### XV.—A CHEAP HOLIDAY FOR NURSES.—UP AND DOWN THE SEINE.

This is a very easy and delightful journey, and may be made so as to suit the most modest purses. There are two ways of reaching the Seine country. I made the journey from Havre to Rouen and back on my machine, and I shall give you that plan, but if you are not cyclists it is quite easy to take the train or to walk the short distances.

### The Havre Route.

This is from Southampton, and is an excellent passage as a rule. Second-class return from Southampton to Havre £1 11s. 8d.; the rest of the journey to be made on wheels; but if you do not cycle the further expense to Rouen and back will be about another 7s.

### The Dieppe Route.

This route straight through to Rouen and back will be £1 13s. 3d., and is therefore a little cheaper, with the additional advantage of being able to stop an hour or two to see Dieppe.

### Expenses for a Fortnight.

These need not exceed £8, including your journey, even if you train all the way. Supposing you remain on French soil fifteen days, spend eight of them at Caudebec, as being the cheaper and also the more delightful resting-place, and seven at Rouen.

### Cyclists First Considered.

The roads are ideal and the distances short, so that the trip commends itself to those not equal to long runs. The people are kind and friendly, and expenses very moderate. If the sufferings of the voyage have not been serious, send on your luggage from Havre and start for Caudebec the same morning, seeing Harfleur, Tancarville, and Lillebonne *en route*. Harfleur will delight the artist and photographer, and as every one possesses a kodak in these days, I feel sure you will enjoy some of the quaint corners of the old town; you must not tarry long, however, but get on to Tancarville, which is in the midst of charming wooded country. Here you will see the ruins of a formidable feudal castle, where one can shiver and tremble in comfortable nineteenth-century-security as one gazes into the dungeons and torture chambers of the "good old days." Go on to Lillebonne and lunch there, visiting the Roman theatre and the castle, starting for Caudebec in the evening; this will make a run of a little under 30 miles.

### Caudebec.

Put up at the Hotel de Marine, where the proprietor is most kind and obliging. Terms seven francs a day by arrangement. By staying at Caudebec and Rouen you can see the whole of the Seine comfortably, and make all the best excursions, either on wheels or on foot, assisted by the rail and the numerous steamers which pass up and down many times a day in the summer. You will soon find out all the excursions, and if you want more information ask me through "Travel Notes and Queries." You must go north, a short run to Yvetot, which makes a nice morning expedition, and the afternoon can be occupied in seeing the ruins of Ste. Wandrille, once an extensive abbey. Pavilly, Duclair, and La Bouille are all near; the little steamer will land you on the other side of the river easily, and the scenery is, I think, on the whole finer on the south bank, the country being more thickly wooded.

### Jumièges and Agnes Sorrel.

Two and a half miles south of Duclair (there is a station there if you are not cycling) is Jumièges, with which you will be delighted; it seems to transport one into the dim "long ago" to stand on that grass-grown abbey pavement and muse on the thousands who once worshipped there and who have long since passed away from this troubled world. How often must the beautiful, lovable, and frail Agnes Sorrel have knelt in these magnificent precincts. She lived at one time at Mesnil-sous-Jumièges, and there she died in the arms of her royal lover, Charles VII., and was buried in the abbey.

### Numerous Excursions.

Besides the places I have mentioned there are dozens of other easy excursions, such as St. Martin de Boscherville, Moulineux, and Elbœuf. Moulineux is to my mind far prettier than La Bouille, but the latter is more popular with artists.

View in Caudebec.

### Rouen.

Go to the Hotel de la Poste; it is an unpretentious place, and will take you on very moderate terms. You can really see Rouen and its environs easily in four days, so that if you prefer to spend still more of your time at Caudebec, you can do so without feeling that you are leaving Rouen unseen. Devote one day to a run down to Elbœuf, where there is an interesting church with unique stained glass. If you have your bicycles go on to Evreux, a distance of 20 miles, but to do that you must be good riders, because remember it means 40 there and back. The Cathedral and Bishop's Palace at Evreux form a celebrated group of architecture. Rouen is a gay city, and you will find omnibuses and trams in all directions; you must walk along the quays to gain an insight into the busy commerce of Rouen. The Cathedral, the churches of St. Owen and St. Maclou are so well known by hearsay if not by sight that I will not stop to speak of them. The Hotel de Ville, once a part of a Benedictine monastery, and the beautiful mansion called Hotel Bougtheroude must be visited, also the Palais de Justice, the "Tour de la Grosse Horloge," and the Tour Jeanne d'Arc. There are some charming walks in the neighbourhood, such as to the church of Bon Secour, distant two miles, and to Boisquillaume. You will find it an agreeable way to return to Havre down the Seine by steamer—fare, second class, 5 fr. 50. You must, however, ascertain at the office, Messrs. Petigny and Bizet, 15, Quay de la Bourse, if the steamers run in connection with the English steamers.

*For Travel Advertisements see page* xvii.

### The Training and Duties of a Norland Nurse.

By One of Them.

Before giving a detailed account of the training at the Norland Institute it would be best to say for what object the institute was intended. It was opened in 1892 to offer a new profession for ladies and to supply the public with trained nurses for young children. The institute began in a very small way, but has rapidly increased, there being now over 200 nurses at work, and the demand for them is great. The training consists of three periods, each period lasting three months, viz.: In the institute, in a children's hospital, in a family.

On the day of arrival at the institute the work is portioned off to the probationers, who are divided into sets of four. They begin their various duties the following day, one set going to a kindergarten school, another to the workrooms, where they are taught to cut out and make up dresses, while a third set is put on domestic duty, when they have a few light household duties to perform. The remaining probationers work together, learning to cut out and make up children's garments. Each set takes a fortnight for these various duties, which only occupy the mornings.

In the afternoons a course of laundry and cookery demonstrations are given. There are also lectures on hygiene and natural history, and brief outlines as to how the various kindergarten occupations should be taught. Soon after four o'clock the probationers stop work for tea, and after lessons go on until dinner is announced.

The evenings pass very pleasantly, as the probationers in it turn sing, read, or recite while the others are working. Saturday is a very easy day; arrears of work have to be made up, then the probationer is free.

When the three months have expired the probationers leave the institute very reluctantly, and go to the various hospitals to receive the second part of the training. There they gain practice in the care of sick children and in noticing signs of illness, &c. This work is rather heavy, and at the end the probationers are glad to have a month's holiday. After this they go to their various posts, and receive their certificates at the end of three months when the training is completed.

The certificate is awarded on the opinion of those responsible for the probationer's work, and not on the results of an examination.

A nurse works for three years, and if at the end of that time her testimonials are satisfactory she becomes a private nurse, when she has many advantages not shared by a nurse.

I can advise girls to take up this work for the following reasons: The training is short, the fees are not at all heavy, the salaries are good, and the work is pleasant on the whole.

### Notes and Queries.

**The contents of the Editor's Letter-box have now reached such unwieldy proportions that it has become necessary to establish a hard and fast rule regarding Answers to Correspondents. In future, all questions requiring replies will continue to be answered in this column without any fee. If an answer is required by letter, a fee of half-a-crown must be enclosed with the note containing the enquiry. We are always pleased to help our numerous correspondents to the fullest extent, and we can trust them to sympathise in the overwhelming amount of writing which makes the new rules a necessity.**

**Every communication must be accompanied by the writer's name and address, otherwise it will receive no attention.**

*Tuberculosis.*

(253) Seeing the account of Dr. Bezly Thorne's lecture to nurses on "The Prevention of Tuberculosis" in The Hospital Supplement, I will be greatly obliged if you will let me know when it appears in pamphlet form, as I am most anxious to secure a copy.—*Masseuse.*

A verbatim report appears in the February number of the *Nurses' Journal*, price 2½d., from the R.B.N.A., 17, Old Cavendish Street, W., and will be published later by Messrs. Churchill.

*The Nightingale Uniform.*

(254) Could you inform me in detail of the uniform worn by the "Nightingale" nurses?—*Glasgow Nurse.*

The uniform of St. Thomas's Hospital—where the Nightingale probationers are trained—is a lavender and white striped washing dress for the probationers, and a blue and white striped one for the staff nurses. The caps are as simple as possible. The sisters have strings to their caps, and wear dark blue sateen dresses.

*Instep.*

(255) A short time ago, when training at a hospital, I got a sharp pain in my foot, and a fellow nurse told me my instep had given way. She advised me to get my shoes blocked. I do not understand what "blocking" means. Is it expensive, and could it be done by any bootmaker, or must it be an anatomical bootmaker? 2. Can you tell me if Pond's arch supports serve the same purpose? I see also that Messrs. Garrould sell shoes fitted with an instep rest. Are these contrivances safe for anyone to wear, or is it better to get one's own shoes "blocked"?—*M. T.*

Ask your nurse friend what she means by "instep giving way" and "blocking." 2. The various instep rests are useful as supports, but they do not cure the evil they are designed to remedy. The great thing in using them, therefore, is to procure a support that fits comfortably, but if you are suffering from "flat foot," other treatment is generally necessary.

*Rheumatism.*

(256) Would you kindly let me know if there is any place for the cure of rheumatism where a nurse would be admitted free? She can get about a little, but has been very much troubled for the last two years with rheumatism in all her joints, and which completely disables her at times. It is very serious in this case, as it is only by nursing she is able to make a living.—*Nurse.*

The Royal Mineral Water Hospital, Bath, is open to the poor of the United Kingdom without recommendation. A medical certificate is required, and certificate of poverty; stay about six weeks. 2. The Devonshire Hospital and Buxton Bath Charity, Buxton, admission by a subscriber's letter. 3. St. John's Brine Baths Hospital for Poor Patients, Droitwich, admission by subscriber's letter, and payment of 5s. weekly. Consult the patient's medical attendant as to the place best suited to the case. Then write to the secretary of the institution he recommends. The patient, from particulars, seems eligible for help from the Hospital Convalescent Fund (if there is a prospect of cure), and for this reason the Droitwich baths are mentioned.

*Radiant Heat Treatment.*

(257) I wish to undergo the Dowsing radiant heat treatment. Can you tell me where I can get this, and be received as a boarder in the same house?—*A. P.*

Your best plan would be to apply direct to Messrs. Dowsing, 24, Budge Row, E.C.

*Weir-Mitchell Treatment.*

(258) Would you kindly inform me of the method of treatment of Dr. Weir-Mitchell, and where I can procure books or pamphlets on the subject?—*Charles E. N.*

Isolation of the patient, prolonged rest, diet, and massage are the chief points in this treatment. "Fat and Blood: An Essay on the Treatment of Certain Forms of Neurasthenia and Hysteria," can be procured from the Scientific Press, London, W., price 5s.

*For Soldiers and Sailors.*

(259) I am a certificated nurse and midwife (holding the L.O.S.), and would like to try for a post in the Hospital for Soldiers' Wives and Children, at Portsmouth. Will you kindly tell me to whom I ought to apply.—*Welfare.*

As there is no hospital of that name at Portsmouth it is possible that you are confusing the Nursing Branch of the Soldiers and Sailors Families' Association with the Royal Portsmouth Hospital. It is the former that supplies district nurses holding L.O.S. to the families of soldiers and sailors at seaports, &c. Apply to the Secretary, 23, Queen Anne's Gate, Westminster.

*Exchange.*

(260) Please tell a nurse with general training, and holding a certificate from Queen Charlotte's Hospital, where she could gain an insight into surgical work on reciprocal terms, or with small salary for her services?—*Nurse H.*

Our advertisement columns might give the information. But a nurse with "general training" has surely had training in surgical work.

## Figures and Tables

**Figure f1:**